# NgR1 knockout increased neuronal excitability and altered seizure pattern in traumatic brain injury mice brain after PTZ-induced seizure

**DOI:** 10.1371/journal.pone.0321447

**Published:** 2025-04-15

**Authors:** Jinwei Zhang, Xin Chen, Kejun Du, Zhi Zhang, Yuan Ma, Yongqin Kuang, Sixun Yu, Haifeng Shu

**Affiliations:** 1 Department of Neurosurgery, General Hospital of Western Theater Command of PLA, Chengdu, China; 2 Department of Neurosurgery, Sichuan Tianfu New Area People’s Hospital, Chengdu, China; George Washington University, UNITED STATES OF AMERICA

## Abstract

The recovery process from traumatic brain injury (TBI) is significantly impeded by inhibitors such as Nogo-A, myelin associated glycoprotein, and oligodendrocyte myelin glycoprotein, which exert an impact on the regeneration and repair of neuronal axons through their binding to Nogo-66 receptor 1 (NgR1). Recent research findings have revealed that NgR1 signaling may play a pivotal role in various seizure mechanisms, including the regulation of synaptic plasticity and migration of neural precursor cells. In this study, wild type (WT) and NgR1 knockout (KO) mice were utilized to establish craniocerebral injury models, while pentylenetetrazol (PTZ) was employed to induce seizures in both groups of mice following TBI. The results revealed that NgR1 KO mice exhibited heightened levels of neuronal electrical activity, along with elevated seizure scores compared to WT controls. Immunofluorescence staining demonstrated an increase in the number of excitatory synapses (P <  0.001) and a decrease in inhibitory synaptic density (P <  0.001) in NgR1 KO mice. Furthermore, the NgR1 KO model mice also displayed an augmentation in the number of presynaptic vesicles (P <  0.001), a narrowing of the synaptic gap (P <  0.001), and an elongation of the synaptic active region (P <  0.001). Our findings have demonstrated that in the previous single cognition of NgR1 inhibition in nerve function repair following TBI, revealing the potential risks associated with inhibiting NgR1 activity in nerve function repair following TBI, and providing a new perspective for understanding the role of NgR1 in the nervous system.

## Introduction

Traumatic brain injury (TBI) is a major cause of death, disability, and mental health disorders. Most patients with TBI suffer long-term posttraumatic stress disorder, cognitive dysfunction, and even epilepsy. Despite the significant medical demand for therapeutic interventions in traumatic brain injury (TBI) patients, there is currently a dearth of pharmacological strategies capable of fundamentally ameliorating TBI and secondary brain damage. This limitation primarily stems from the intricate challenges associated with the central nervous system (CNS) remodeling and repairing following trauma, wherein the presence of axon growth inhibitors, including myelin-associated glycoproteins, oligodendrocyte myelin glycoprotein, and Nogo-A, emerges as a pivotal factor [1]. These proteins, all bind to the same neuronal glycosylphosphatidylinositol-anchored receptor NgR1, and transduce inhibitory signals to cells by the transmembrane co-receptor, p75. The final outcome of this event is inhibition of axonal regeneration and recovery of locomotion [2]. Based on these findings, NgR1 has garnered attention as a potent inhibitor of axonal regeneration following myelin-associated CNS injury and represents a promising therapeutic target.

In both rodents and non-human primates, pharmacological inhibition of NgR1 using NEP1–40 effectively disrupts the interaction between NgR1 and its ligand, resulting in significant axonal growth within the corticospinal tract and improved CNS injury [3–5]. Similarly, inhibition of NgR1 has demonstrated promising and appealing results in promoting neurological function recovery across various neurological disorders including Alzheimer’s disease [6], ischemic stroke [7], optic nerve injury [8], and multiple sclerosis [9]. With the advancement of research, an increasing array of biological functions associated with NgR1 signaling have been unveiled. However, when considering the utilization of NgR1 inhibition for therapeutic purposes in pathological conditions, it is imperative to meticulously evaluate potential concomitant adverse effects so as not to disregard latent risks in pursuit of benefits. For example, the role of NgR1 in the modulation of synaptic plasticity has been demonstrated. Studies of hippocampal slice long-term potentiation (LTP) demonstrated that administration of soluble Nogo-66 or OMgp altered synaptic plasticity, suppressing LTP and enhancing LTD [10, 11]. These effects were dependent on the presence of NgR1 as a receptor. In separate studies, acute neutralization of endogenous NgR1 increased LTP [12, 13]. These studies suggest that inhibition of NgR1 can enhance synaptic plasticity, creating a favorable environment for the recovery of neural function after CNS injury [14–16]. However, the regulation of synaptic plasticity and structure is thought to impact on the neuroelectrophysiological network, potentially playing a role in the development of epilepsy under specific pathological circumstances.[17]. Furthermore, the involvement of the NgR1 signaling pathway in the modulation of neurotransmitters necessitates meticulous deliberation. In an early study, silencing of either Nogo-A or NgR1 was shown to increase the levels of the post-synaptic density protein PSD95, as well as AMPAR (GluA1/GluA2) and NMDAR (GluN1/GluN2A/GluN2B) subunits [18].On the other hand, changes in neuronal activity can also affect the expression of the NgR1 receptor. In the adult neocortex and hippocampus, neuronal activity regulates the promoter of NgR1, and an increase in neuronal activity induced by kainic acid leads to a rapid downregulation of NgR1 mRNA expression [19]. Studies involving human brain tissue have shown that epileptic foci exhibit significantly lower levels of NgR1 compared to normal brain tissue [20]. These studies confirm a strong correlation between neural network activity and NgR1 signaling. At present, it is known that neuronal network remodeling after TBI may lead to the formation of local excitatory networks and even lead to epilepsy, but the possible effects of NgR1 targeted intervention on neural networks after TBI have not been reported. Hence, it is imperative to investigate the potential impact of NgR1 loss on neuroelectrophysiology in individuals suffering from TBI.

To investigate the impact of NgR1 on brain electrophysiology after TBI, we examined the electrophysiological activity in NgR1 KO mice using the Controlled Cortical Impact (CCI) model. Furthermore, we induced seizures in mice using PTZ based on the CCI model to evaluate the effects of NgR1 KO on seizure severity and subsequent behavioral changes resulting after TBI. Then, we investigated alterations in both excitatory and inhibitory synapses utilizing immunofluorescence double labeling colocalization techniques, while also examining the synaptic substructure using TEM. This study, for the first time, demonstrates that NgR1 gene deficiency exacerbates susceptibility to PTZ-induced epilepsy following TBI by increasing excitatory synaptic density and altering synaptic ultrastructure. These findings suggest that therapeutic strategies targeting NgR1 need to carefully balance neural repair and epilepsy risk.

## Materials and methods

### Animals

All animal use protocols were reviewed and approved by the General Hospital of Western Theater Command(2023ky149-1) and performed in accordance with the international guidelines on the use of laboratory animals. NgR1 KO- C57BL/6 mice obtained from Cyagen Biosciences (Suzhou, China) and mating and breeding of their offspring in Chengdu Dossy Experimental Animals Co., Ltd. Housed under laboratory conditions (relative humidity of 45–55%, 12-h light/dark cycle, freely available food and water) at room temperature of 20–23 °C, and laboratory technicians conducted daily checks. The experimental mice had a weight range of 20–22 grams and were 8- to 10-week-old and the NgR1 KO-C57BL/6 mice necessitate genetic testing. Genotyping was performed using polymerase chain reaction (PCR) with the following primers: mutant forward, TCATGTCAGCAGGTGTATGAGGTAG; mutant reverse, GAAGGAGATGCTATGCTGGGAC; WT forward, GAGTAGAAAGCAGATGTCCTCCATC; and WT reverse, CATTAGAGTGCAGCCACAGGATAG. As shown in S1a Fig, a 512-bp PCR band was detected for WT mice, 512- and 735-bp products were detected for heterozygous animals, and a single 735-bp product was detected for homozygous mice.

### Controlled cortical impact (CCI) procedure

The CCI model is widely utilized in mice as one of the most prevalent TBI models, enabling the induction of TBI across a spectrum from mild to severe by precisely regulating the depth of impact. CCI procedure followed the protocol as previously described [21]. Mice were anesthetized with isoflurane and fixed in stereotaxic frame. After the skull was exposed, an ∼ 4-mm diameter craniotomy was made to the right of the sagittal suture centered between bregma and lambda with the medial edge 2 mm lateral to the midline [22]. The controlled contusion device (Chinese Utility Model Patent; patent nos. ZL 2017 2 1543642.1 and 2017 2 1542057.X) consisted of a computer-controlled, electrokinetically driven impactor fitted with a 2-mm-diameter flat stainless-steel tip. Injury was induced using this device by compressing the cortex to a depth of 1.0 mm (hard stop) at a velocity of 3.5 m/sec and for a duration of 100 msec. After impacting the cortex, local hemostasis was applied. Then, the bone flap was repositioned and the scalp was sutured before placing it in a dedicated recovery box. On days 1, 3, 7 and 14 post-CCI, mice that had succumbed to various causes were excluded from the study and the Mouse Revised Neurobehavioral Severity Scale (mNSS-R) [23]was employed to evaluate motor, sensory, and reflex functions in NgR1 KO and wild-type mice (S1 Fig). Mice (2 in WT group and 3 in NgR1 KO group) died before completing the experimental test. The group, number, and time of death are provided in S1 Table.

### Video electroencephalogram (EEG) recording and PTZ-induced seizure susceptibility

At 2 weeks post TBI, a plastic electrode pedestal with six silver wire electrodes was implanted as shown in (S2b Fig). Four epidural electrodes were implanted into the position of 1 and 5 mm anterior to lambda and 4 mm lateral to the midline over each hemisphere, one silver wire electrode was placed in the cerebellum as a quasi-reference electrode, and the last silver wire electrode served as a ground wire. After a recovery period of 7 days, the mice underwent EEG recording and video monitoring with a signal collecting and processing system (RM6240EC; Chengdu Instrument Factory, Chengdu, China) under free moving. After 5 min of habituation, continuous EEG monitoring was performed for at least 10 min. To validate seizure susceptibility, we administered a non-convulsant dose of PTZ (30 mg/kg, i.p, Sigma-Aldrich, St. Louis, MO, USA) [24], a CNS stimulant that acts on the γ-aminobutyric acid (GABA)A receptor, to induce seizures, and video EEG recording was performed for 60 min.

### Video EEG and Seizure classification

Clinical seizure score were assigned as follows as described previously [25]: 0) normal behavior (no abnormality); 1) facial twitching and freezing; 2) neck jerks and head nodding; 3) myoclonic jerks, forelimb clonus, and tail rearing; 4) tonic seizures and falling down on the side; 5) tonic-clonic seizures, falling down on the back, wild rushing, and jumping; and 6) death. During EEG preprocessing, the raw EEG data were low-pass filtered at 50 Hz, and artifacts were removed with a homemade algorithm developed in MATLAB (MathWorks Inc., Natick, MA) [26]. A spike was defined as a high-amplitude (twice the baseline amplitude) sharply contoured waveform with a duration of 20–70ms. An electrographic seizure was defined as high-amplitude rhythmic discharge with a duration of > 5 s and a clear onset. The results were manually reviewed by a team of neurophysiologists who are certified by the Health Commission of the People’s Republic of China and have years of experience in practicing neurophysiology at the General Hospital of Western Theater Command of PLA. Video recordings were viewed to identify seizures and to assess behavioral changes during the electrographic seizures.

### EEG analysis

The EEG power patterns were detected using Morlet wavelet analysis and represented through spectral density heatmaps. For quantitative analysis of the spectral density heatmaps, we adopted the root mean square (RMS) analysis method as proposed by Kevin D et al [27,28]. RMS is a statistical measure used to assess the strength of biological signals and provides an estimation of their magnitude. The RMS is a statistical measure of the magnitude of a varying quantity. It can be calculated for a series of discrete values or for a continuously varying function. RMS is one of the most commonly used methods that measure the amplitude of a bio- signal, e.g., EEG signals and electromyographic signals. The amplitude of a bio-signal expresses the magnitude of the power (energy per time) of that particular signal. We used Brainstorm and customized MATLAB code to analyze RMS power in the background and seizures episodes [29]. The RMS power values for the delta (0.5–4Hz), theta (4.5–7.5Hz), alpha (8–13Hz), beta (14–30Hz) and gamma (35–45Hz) frequency bands were calculated in one-minute intervals by averaging the values of 15 four-second windows. Noting that only the ipsilateral EEG data were used for subsequent Morlet wavelet analysis and RMS power analysis (S2c Fig). After video EEG monitoring, specifically three weeks post-CCI, the mice reached the clinical endpoint and were euthanized by decapitation performed by professional technicians. Subsequently, their brains were removed.

### Immunofluorescence (IF) staining

After video EEG monitoring, the mice were anesthetized with isoflurane and subsequently underwent perfusion, fixation, dehydration, and embedding, as previously described [30]. 5 μm thick frozen coronal slices of cortex were prepared from CCI-injured mice (Microm H M 525 cryostat, Thermo Fisher Scientific), from cortical regions ipsilateral and directly adjacent to the injury site (1.5–2.0mm rostral and/or caudal). For fluorescence labeling, free-floating slices were rinsed in 0.01 M PBS (pH 7.4) and blocked with 0.5% Triton-X 100 and 10% normal goat serum for 60 min at 37°C, then incubated overnight at 4°C with the following primary antibodies: rabbit anti-VGLUT1 (1:500, GTX133148, GeneTex, USA), mouse anti-PSD95 (1:500, 6G6-1C9, Thermo Fisher Scientific, USA), rabbit anti-Gephyrin (1:1000, GTX109734, GeneTex, USA), mouse anti-VGAT (1:150, NBP2–46628, Novus Biologicals, USA), and at 37°C for 90 min with the following secondary antibodies: CY3-labeled goat anti-rabbit IgG (1:400, BA1032, BOSTER, China) and FITC-labeled goat anti-mouse (1:2000, ab6785, Abcam, USA). The techniques for acquiring and analyzing synaptic number through fluorescence primarily draw upon the research findings of Dominic M et al [30]. Fluorescence signals were detected and imaged using a Nikon A1R confocal microscope (Nikon, Japan) under the 100x oil objective lens (1.45 numerical aperture), 2,048 ×  2,048-pixel format. The image acquisition and analysis were blinded to the experimenter. For each brain slice, a total of 15 optical slices were imaged at 0.33 μm intervals at a total depth of 5 μm in consecutive optical slices. Maximum image projections (MIPs) were generated for groups of 3 consecutive optical sections yielding 5 MIPs/section each representing 1 μm of depth. Regions of interest (ROI) were selected from target regions, including the cerebral cortex adjacent to the site of trauma, i.e., the ipilateral and the cortical regions directly adjacent to the site of injury (specifically the 1.5 - to 2.0 mm rostral and/or caudal sides), with a particular focus on layer V cortex. The same light intensity and exposure settings were used for all slices within each image set. Quantification of co-localized synaptic puncta was performed using ImageJ-puncta analyzer to select the ROI, Synaptic puncta quantification was presented as density like synaptic puncta counts/400 mm2. Set the minimum puncta size to 4 pixels. There were 3 mice per group, and 5 sections/animal and 4–5 images of each section were analyzed.

### Transmission electron microscopy (TEM)

After the mice were sacrificed by decapitation, the brains were removed, the ipsilateral cortex was dissected and washed in 0.1 M phosphate buffer (pH 7.4). the tissue blocks were then fixed with 1% osmium tetroxide in 0.1 M PB (pH 7.4) for 2 h at room temperature, and routinely dehydrated using graded ethanol. From cortical regions ipsilateral and directly adjacent to the injury site (1.5–2.0mm rostral and/or caudal), the resin blocks were cut into 70nm thick sections on an ultramicrotome after resin penetration and embedding, and the tissues were stained with 2% uranium acetate saturated alcohol solution, 2.6% lead citrate and finally observed under a transmission electron microscope (HT7800/HT7700, HITACHI, Japan) at 6,000 × and 15,000 × magnification. The number of synaptic vesicles, length of the synaptic active zone, synaptic cleft width, postsynaptic density (PSD) thickness and synaptic curvature were measured using Fiji software as described previously [31].

### Statistical analysis

Statistical analysis was performed using Prism 8 (GraphPad Software). The results were presented as mean ±  standard error (SEM). RMS power analysis were performed with two-way ANOVA with Holm-Šídák test post hoc analysis. 7 mice in each of the WT and NgR1 KO groups were used for RMS power analysis (n = 7); Student’s t-test was employed to compare epileptic susceptibility, synaptic number, and synaptic interface parameters. The resulting n’s for PTZ-induced epileptic susceptibility experiments are n = 17 for WT, n = 15 for NgR1 KO. For synaptic number counting, there were 3 mice per group (n = 3), and 3 sections/animal and 4–5 images of each section were analyzed. There were 3 mice in each group, and 3 images/animal were analyzed for ultrastructural analysis. Significance levels were set at * p <  0.05, **p <  0.01, ***p <  0.001.

## Results

### The NgR1 KO mice exhibited a significant alteration in the post-traumatic EEG background, as well as in the EEG after PTZ-induced epilepsy

**Fig 1:**
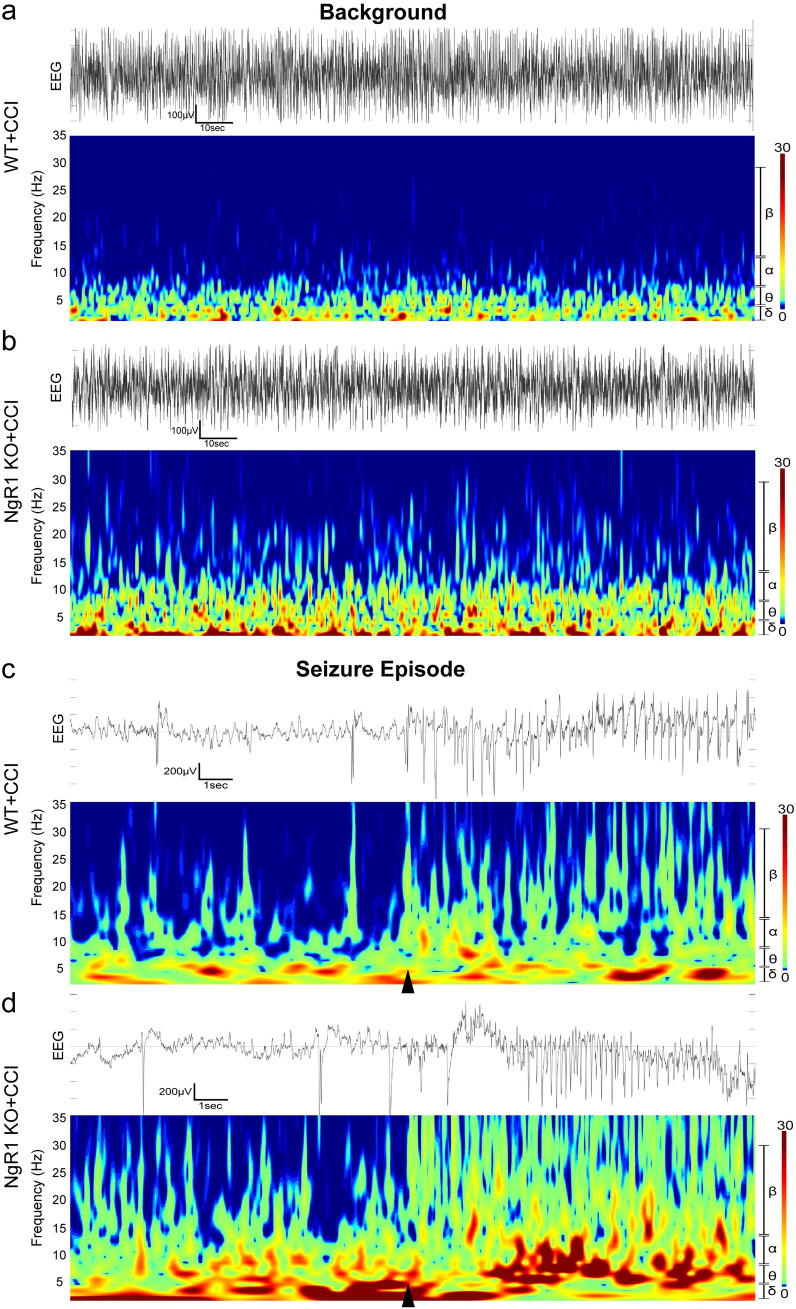
Comparison of spectral characteristics in the interictal background and PTZ-induced epileptiform discharges in WT and NgR1 KO mice. The EEG signals and spectral heatmap of a representative WT mice and NgR1 KO mice are shown and the vertical axis of the spectral heatmap displays the frequencies, which are categorized into 5 bands (δ, θ, α, β, γ), with corresponding labels presented on the right vertical axis. In addition, on the right scale bar, warm colors represent higher power, and cool colors represent lower power. (a) From the range and concentration of warm colors in the background, it is evident that WT mice exhibit predominant brain electrical power at lower frequencies (δ, θ). (b) The NgR1 KO mice, however, also displayed more pronounced electrical activity in the α, β, and γ frequency bands compared to WT mice, in addition to δ, θ bands, indicating a greater overall level of electrical activity. Scale bars for the EEG plot: 100 μV, 10 sec. (c, d) Representative EEG power in NgR1 KO and WT mice during SE onset. Scale bars: 200 µ V, 1 sec. The start of the SE is marked by a black triangle. After PTZ injection, both NgR1 KO and WT mice exhibited a sharp increase in EEG activity across all frequencies (δ, θ, α, β, γ) at status epilepticus (SE) initiation (black triangle), and the spectral heatmap reveals a more pronounced warm-color range of EEG activity in NgR1 KO mice compared to WT mice. Furthermore, NgR1 KO mice exhibited greater enhancement of EEG activity in the α band frequency than WT mice. In contrast, the EEG activity of WT mice was mainly concentrated in the δ and θ bands.

Initially, we utilized WT and NgR1 KO mice to establish the CCI model, and conducted mNSS-R. The results showed that NgR1 KO and WT mice had no difference in neural function deficits after CCI (S1b Fig and S2 Table). Mice underwent electrode implantation 2 weeks after CCI. Subsequently, EEG recordings were performed after a 7-day waiting period. The spectral density heatmaps were utilized to visualize the background and PTZ-induced changes in EEG activity, employing the Brainstorm software based on Morlet wavelet analysis. In the background, from the range and concentration of warm colors, it is evident that WT mice exhibit predominant brain electrical power at lower frequencies (δ, θ) (Fig 1a). The NgR1 KO mice, however, also displayed more pronounced electrical activity in the α, β, and γ frequency bands compared to WT mice, in addition to δ, θ bands, indicating a greater overall level of electrical activity (Fig 1b). After PTZ injection, both NgR1 KO and WT mice exhibited a sharp increase in EEG activity across all frequencies at status epilepticus (SE) initiation (black triangle), and the increase in EEG activity was more pronounced in NgR1 KO mice than WT mice (Fig 1c, d). Furthermore, NgR1 KO mice exhibited greater enhancement of EEG activity in the alpha band frequency than WT mice. In contrast, the EEG activity of WT mice was mainly concentrated in the δ and θ bands. Thus, a reduction in NgR1 levels induced changes in EEG activity prior to PTZ-induced seizures, and NgR1 KO mice exhibited different EEG activity patterns in response to TBI than WT mice. In addition, an important control was observed, that is the EEG of WT and KO animals without TBI, we found that no significant difference in background EEG between WT and NgR1 KO mice, which was shown in the S3 Fig.

**Fig 2:**
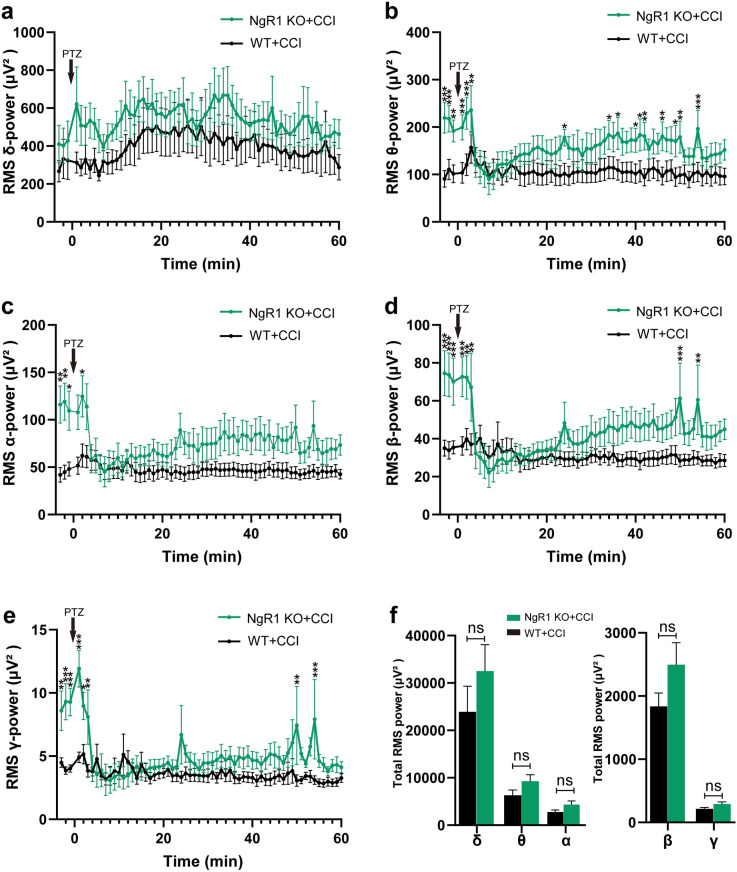
The changes of EEG activity in different frequency bands. The time course of RMS power analysis including the delta (a), theta (b), alpha (c), beta (d), and gamma (e) bands in WT and NgR1 KO mice 3 min before and 60 min after administration of PTZ; the arrow indicates injection of PTZ (30 mg/kg, i.p). Before PTZ injection (delimit by PTZ black arrow), the baseline of NgR1 KO mice was higher than that of WT mice in all frequency bands except δ bands(a-e). Similarly, after PTZ induced seizures, RMS analysis showed that NgR1 mutant mice had higher baseline values in all bands except δ than WT mice, with β, γ, and θ bands being the most significant (b, d, e), electrical activity in both groups then fell silent for several minutes. (n = 7 mice, * P < 0.05; **P < 0.01; ***P <  0.001, two-way ANOVA, the error bars represent the SEMs). (f) Total RMS Power within 60-minute timeframe after PTZ-injection across five frequency bands (ns, not significant).

To further clarify the above conclusions, we used Brainstorm software and custom MATLAB code to perform RMS power analysis, aiming to quantify each frequency band (δ, θ, α, β, γ) in the above spectral density heat map. The results of our analysis indicated that NgR1 mutant mice exhibited a completely different firing pattern (Fig 2). First, before PTZ injection (delimit by PTZ black arrow), the baseline of NgR1 KO mice was higher than that of WT mice in all frequency bands except δ, and the analysis results exhibited statistical significance (Fig 2a-e, **P <  0.01; ***P <  0.001). Similarly, after PTZ induced seizures, RMS analysis showed that NgR1 mutant mice had higher baseline values in all bands except delta than WT mice, with β, γ, and θ bands being the most significant (Fig 2b, d, e.***P <  0.001), electrical activity in both groups then fell silent for several minutes. These results suggest that NgR1 KO mice exhibit a completely different RMS power pattern, showing a more active and excited EEG background in resting state than WT mice, and a more severe seizure degree and stronger EEG energy release a following PTZ. This aligns with the spectral density heatmaps in Fig 1. From the RMS analysis diagram, it was observed that the power fluctuations in each frequency band after PTZ-injection. There was a gradual increase in baseline levels within 60 minutes after a sharp decline post-seizure. Consequently, we computed the total RMS Power within this 60-minute timeframe after PTZ-injection across five frequency bands. However, no statistically significant difference was found within this duration (Fig 2f), which could potentially be attributed to the silent period occurring after seizures.

### NgR1 mutant mice exhibit increased seizure score and neuronal electrical activity in response to PTZ-induced seizures after TBI

**Fig 3:**
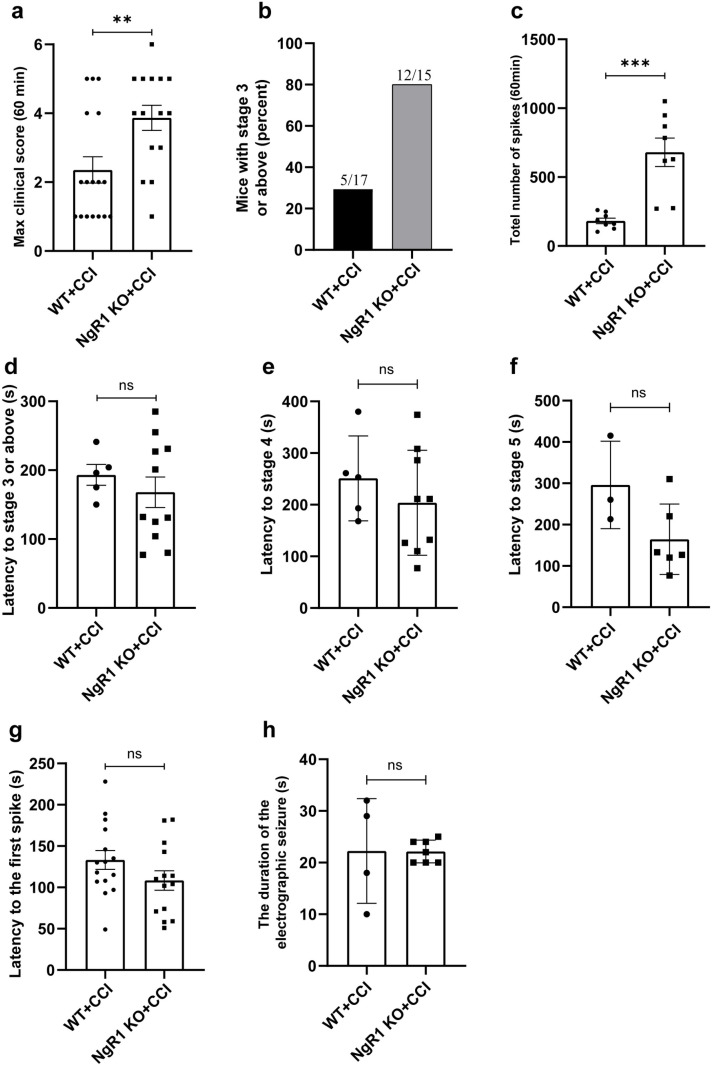
NgR1 mutant mice exhibit increased susceptibility to PTZ-induced seizures after CCI. Seizure severity was assessed by determining the maximum clinical epileptic score (a) and the number of NgR1 KO and WT mice with myoclonic jerks (stage 3) or above seizures (b) within 60 min following PTZ administration. (a) In CCI model mice received PTZ to induced seizure, the clinical seizure score of NgR1 KO mice was significantly increased compared with WT group, and the difference was significant (WT mice, n = 17; NgR1 KO mice, n = 15. **P = .0079). (b) Among the mice assessed, a significantly higher proportion of NgR1 KO group (12/15) exhibited grade 3 seizures compared to the WT group (5/17). (c) The total number of spikes of NgR1 KO group within 60 min following PTZ was significantly increased compared with WT group (n = 8, ***P = .0003). (d) The NgR1 KO group did not exhibit a significant difference compared to the WT group in terms of the latency to myoclonic jerks (stage 3) seizures. (WT mice, n = 5; NgR1 KO mice, n = 11). (e) The NgR1 KO group did not exhibit a significant difference compared to the WT group in terms of the latency to myoclonic jerks (stage 4) seizures (WT = 5, NgR1 KO = 9). (f) The NgR1 KO group did not exhibit a significant difference compared to the WT group in terms of the latency to myoclonic jerks (stage 5) seizures (WT = 3, NgR1 KO = 6). (g) The latency of the initial peak did not exhibit a statistically significant difference between the NgR1 KO group and WT group (WT mice, n = 15; NgR1 KO mice, n = 14). (h) The duration of the electrographic seizure did not exhibit a statistically significant difference between the NgR1 KO group and WT group (WT = 4, NgR1 KO = 7). (ns, not significant); unpaired Student’s t-test. The error bars represent the SEMs.

In CCI model mice received PTZ to induced seizure, the clinical seizure score of NgR1 KO mice was significantly increased compared with WT group, and the difference was significant (Fig 3a, b. *  *  P <  0.01). For quantitative analysis of EEG spikes, we found that NgR1 KO group increased the total number of spikes within 60 min after PTZ injection compared to the WT group (Fig 3c. ***P <  0.001). However, NgR1 KO did not alter the latency to myoclonic jerks (stage 3) or the latency to the first spikes (Fig 3d, g). Similarly, we calculated the latency to the first stage 4/5 seizure of WT and NgR1 KO mice as shown in the figure below (Fig 3e, f). There was no statistical difference between WT and NgR1 KO mice in latency to stage 4/5 seizures. Additionally, we analyzed the duration of the electrographic seizure (Fig 3h) and found no statistical difference between the two groups.

### NgR1 KO mice exhibit more excitatory synapses than WT mice and alterations in the ultrastructure of synaptic endings

**Fig 4:**
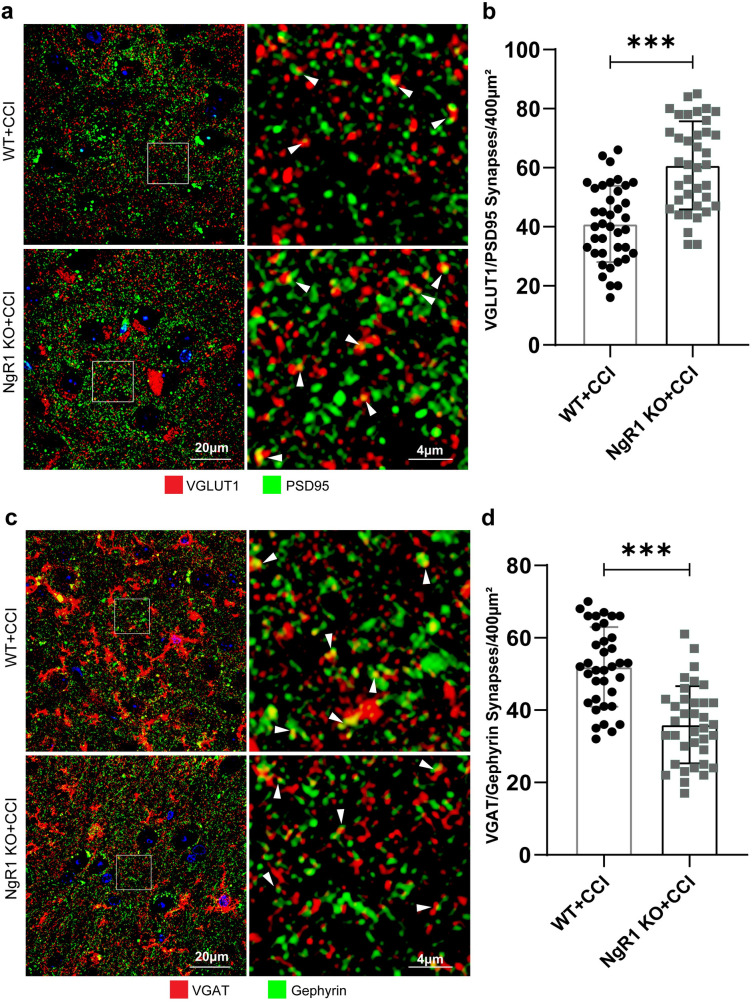
NgR1 KO mice exhibited an increase in excitatory synapses and a decrease in inhibitory synapses, indicating an imbalance of synaptic transmission. (a) Representative IF staining of VGLUT1 (red) and PSD95 (green) in the ipsilateral cortex in slices from WT and NgR1 KO mice. Colocalization of these proteins is synaptic puncta is indicated by yellow puncta (arrowhead). (b) Blinded analysis of VGLUT1/PSD95 colocalization in cortical sections from WT and NgR1 KO mice. The excitatory synaptic density of NgR1 mice showed a significant increase, with statistically significant differences observed when compared to WT mice (***P <  0.001). (c) Representative IF staining of VGAT (red) and Gephyrin (green) in the ipsilateral cortex in slices from WT and NgR1 KO mice. (d) Blinded analysis of VGAT/Gephyrin colocalization in cortical sections from WT and NgR1 KO mice. The inhibitory synaptic density of NgR1 mice showed a significant decrease when compared to WT mice (***P <  0.001). There were 3 mice per group, and 5 sections/animal and 4-5 images of each section were analyzed. ***P <  0.001. Scale bar: 20 μm.

A reduction in the levels of NgR1, as a synaptic regulator, promotes new dendritic spines in vitro, and these new dendritic spines are functional and involved in the integration of the nervous system [32]. Are these new dendritic spines involved in the excitation/inhibition imbalance? In our study, sections from WT and NgR1 KO mice were double-immunolabeled for presynaptic (VGLUT1) and postsynaptic (PSD95) excitatory synapses, and VGLUT1 (red)- and PSD95 (green)-positive puncta (yellow) were presumed to represent synaptic contacts (Fig 4a). Similarly, synaptic contacts are identified by the co-localization of VGAT (red) and Gephyrin (green), which label inhibitory synapses, resulting in yellow positive spots (Fig 4c). A blinded analysis of colocalization showed that the excitatory synaptic density showed a significant increase, while the inhibitory synaptic density exhibited a notable decrease in NgR1 KO mice following cortical injury, with statistically significant differences observed when compared to WT mice. (Fig 4b, d. ***P <  0.001).

### The ultrastructural alterations of synapses were observed in NgR1 KO mice and WT mice after TBI

**Fig 5:**
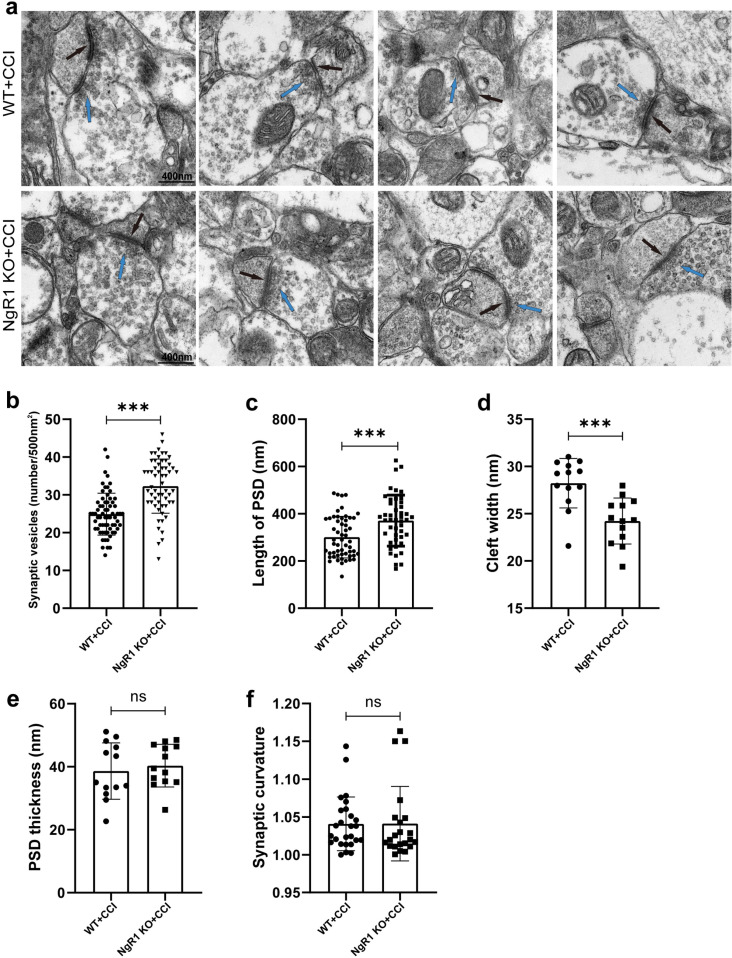
Ultrastructural alterations in the cerebral cortex following TBI in NgR1 KO and WT mice. (a) The synaptic terminal ultrastructure of WT and NgR1 KO mice, such as synaptic vesicles as indicated by the black and blue arrow (Scale bar: 400 nm). Compared to the WT group, NgR1 KO mice exhibited a significant increase in presynaptic vesicles (b, ***P <  0.001), an elongated PSD (c, ***P <  0.001), and a narrower gap width (d, ***P <  0.001), while no differences were observed in synaptic curvature and PSD thickness (e, f). There were 3 mice (n = 3) in each group, and 3 images/animal were analyzed; unpaired Student’s t-test.

To further investigate the ultrastructural changes of synapses, sections were carefully selected from both the ipsilateral and cortical areas directly adjacent to the injury site (1.5–2.0mm rostral and/or caudal), followed by evaluation of synapse ultrastructure using TEM (Fig 5a). There were 3 mice in each group(n = 3), and 3 images/animal were analyzed. Compared to the WT group, NgR1 KO mice exhibited a significant increase in presynaptic vesicles (Fig 5b ***P <  0.001), an elongated PSD (Fig 5c ***P <  0.001), and a narrower gap width (Fig 5d ***P <  0.001), while no differences were observed in synaptic curvature and PSD thickness (Fig 5e, f).

## Discussion

This report first found higher baseline EEG activity in NgR1 KO mice than in WT mice after CCI, indicated NgR1 KO mice have greater neuronal excitability than WT mice after TBI. Subsequently, TBI mice performed PTZ seizure susceptibility test, NgR1 KO mice exhibited a much worse clinical epileptic seizure response to PTZ-injection and a more drastic energy release occurred. These results suggest that in the pathological context of TBI, NgR1 deficiency may lead to abnormal excessive or synchronous neuronal activity during stress recovery in brain.

The reasons for this result are worth exploring. Previous studies have confirmed that in non-pathological conditions, the brains of NgR1 mutant mice do not show significant defects at the anatomical level and no significant changes in spine density and net excitatory synaptic activity in the hippocampus [11], which is associated with an experience-driven “braking” mechanism of neuroplasticity after development to a mature brain. However, it has been observed that NgR1-deficient mice show persistent excitatory synaptic AMPAR transmission [33]. So, would NgR1-deficient ones similarly affect synaptic alterations in the stressful situation of TBI? In our study, we observed changes in excitatory synapses, and our in vivo studies showed that NgR1-deficient TBI mice had an increased number of cortical excitatory synapses, which is consistent with previous literature that NgR1 deficiency leads to a significant increase in the number of excitatory synapses in cultured hippocampal neurons [32].

Do these increased excitatory synapses have a functional basis for increasing neuronal excitability? We simultaneously observed the ultrastructure of synaptic endings using TEM and analyzed synaptic structural parameters including synaptic vesicle number, length of the PSD, cleft width, PSD thickness and synaptic curvature, which is thought to have an important influence on the speed and effectiveness of chemical synaptic transmission [34], Our study revealed that NgR1 KO mice possess a greater number of presynaptic vesicles than WT mice, which may lead to increased neurotransmitter release and subsequent synaptic transmission. We also observed that NgR1 KO mice exhibited a narrower cleft width and a longer PSD (a longer synaptic active zone) than WT mice, which may occur secondary to the increase in presynaptic vesicle number to meet the need of increased efficacy of synaptic transmission and reflect improvements in the speed and efficiency of chemical synaptic transmission, may involved in enhanced neuronal activity.

The subsequent section will address the limitations of the present study. First of all, we used the epileptic agent PTZ based on previous studies [35,36], which antagonizes the GABA_A_ receptor and causes neuronal excitability. PTZ-induced epilepsy does not represent the underlying mechanism of post-traumatic epilepsy and therefore cannot serve as a representative model for studying secondary epilepsy following traumatic brain injury. However, it may offer some insights into the impact of NgR1 KO on seizure activity to a certain extent. Future research should focus on exploring more appropriate models for studying post-traumatic epilepsy. Secondly, our investigation into the impact of NgR1 KO on synapse numbers revealed an augmentation in excitatory synapses and a reduction in inhibitory synapses. This phenomenon appears to elucidate the underlying mechanism behind the heightened EEG activity and increased seizure severity induced by CCI-PTZ following NgR1 KO. However, it is crucial to acknowledge that Nogo-A, the upstream ligand of NgR1, regulates synapses in a more intricate manner. Previous studies have demonstrated that activation of different receptors by Nogo-A signals can modulate the expression and dynamics of both excitatory and inhibitory neurotransmitter receptors [37]. The Nogo-A-NgR1 signaling pathway is implicated in regulating excitatory neurotransmitter release, while abrogating or diminishing NgR1 function enhances excitatory synaptic transmission. On the other hand, Nogo-A-S1PR2 signaling alters GABAAR stability through inhibition of calcium influx, thereby affecting inhibitory neurotransmission. Our study did not investigate feedback regulation from other Nogo-A receptors after NgR1 knockout, so the mechanism behind NgR1 KO increased nervous system excitability may be more complex than we currently understand. Therefore, our current study adds to existing knowledge by revealing only one aspect - that NgR1 signaling influences both types of synapses under stress conditions such as trauma. Furthermore, we were not able to provide direct evidence for the correlation between NgR1 KO and posttraumatic epilepsy. Nevertheless, the results of this experiment also give us new hints, as NgR1 KO mice showed excessive plasticity, leading to impaired consolidation of newly formed synapses and circuits, and they may respond more vigorously to stimuli, thus showing greater neuronal excitability in response to PTZ. Therefore, the use of neurostimulant drugs should be avoided when antagonizing NgR1 as a treatment strategy. On the other hand, we hypothesize that more aggressive treatment is given in response to increased plasticity, and the results may be much more positive. We speculate that increased NgR1 expression in patients with epileptic seizures or SE can limit synaptic plasticity and slow the formation of abnormal synaptic connections and abnormal neuronal circuits by inducing multiple seizures.

Despite these limitations, this study yields several significant findings that will inform future research on the potential of NgR1-related pathways in craniocerebral trauma treatment. Most notably, this study pioneers the evaluation of whether NgR1 deficiency can induce heightened excitability of cortical nerves following traumatic brain injury and its impact on PTZ-induced epilepsy’s electrophysiology. Furthermore, it confirms a close association between these changes and NgR1’s involvement in regulating excitatory synapses and synaptic ultrastructure, thereby providing novel insights into the role of myelin inhibitors in post-traumatic brain injury epilepsy occurrence.

## Conclusions

Our study indicates that NgR1 signaling has a dual role in regulating neuroexcitability following TBI: its inhibition may promote axonal regeneration but may also increase epilepsy risk through an imbalance of excitatory/inhibitory synapses. Therefore, the development of NgR1-targeted therapies should consider the timing of intervention and compensatory mechanisms.

## Supporting information

S1 Fig (a) PCR identification results for a portion of mice. Among them, a single 512-bp band represents wild-type mice, the presence of both 512-bp and 735-bp bands indicates heterozygosity, and a single 735-bp band signifies homozygosity. (b)Mouse Revised Neurobehavioral Severity Scale of WT and NgR1 KO mice were performed at 1, 3, 7 and 14 days after CCI. (c) The results showed that the severity of neurological function loss was the highest at 1 day after traumatic brain injury. After that, the neurological function gradually improved. There was no significant difference in Neurobehavioral Severity Scale between the two groups (n = 9, two-way ANOVA).
(TIF)

S2 Fig (a)photomicrograph of an immunohistochemically stained coronal section from an NgR1 KO mouse (#13), which was sacrificed 2 weeks after CCI. The black arrow indicates the injury site. (b) Diagram of cortical electrode implantation. (c) Primary EEG signal obtained from the 4 electrodes. Electrode 1ch and electrode 3ch represent ipsilateral electrodes (injured side of the brain). Electrode 2ch and electrode 3ch collected contralateral cortical EEG signals. Note that only the ipsilateral EEG data were used for the subsequent Morlet wavelet analysis and RMS power analysis.
(TIF)

S3 Fig Comparison of spectral density heatmaps and RMS analysis of the EEG background in WT mice and NgR1 KO mice without CCI. (a) The EEG signals and spectral heatmap of a representative WT mice and NgR1 KO mice are shown and the vertical axis of the spectral heatmap displays the frequencies, which are categorized into 5 bands (δ, θ, α, β, γ), with corresponding labels presented on the right vertical axis. In addition, on the right scale bar, warm colors represent higher power, and cool colors represent lower power. (a) From the range and concentration of warm colors in the background, it is evident that WT mice and NgR1 exhibit predominant brain electrical power at lower frequencies (δ, θ); (b) Power analysis including the δ, θ, α, β, γ bands in WT and NgR1 KO mice. The RMS analysis revealed that the baseline power in the δ, θ frequency bands of NgR1 KO mice was lower compared to WT mice. Conversely, the baseline power in the α, β and γ frequency bands was higher in NgR1 KO mice than in WT mice. However, there were no statistically significant differences observed in δ, α, β, and γ when considering the overall statistics. Only in the θ band did NgR1 KO mice exhibit lower baseline power compared to WT mice, with statistical significance. (ns, non-statistical significance; * P <  0.05).
(TIF)

S1 Table Mortality in WT and NgR1 KO mice after CCI and after PTZ test. The injury-related mortality rate within the 24 h post-CCI was 5.3% (1/19) in the WT-CCI group and 5.9% (1/17) in the NgR1 KO-CCI group, with both deaths attributed to subdural hematoma. Follow-up mNSS-R evaluations were performed on postoperative day 1、3、7、14 to assess neurobehavioral dysfunction. 1/16 mice (6.3%) in the NgR1 KO-CCI group died in 4–7days post-CCI (euthanized due to poor wound healing resulting in decapitation), and 1/18 mice (5.6%) in the WT-CCI group died in 8–14 days post-CCI (cause of death unknown). 1/15 mice (6.7%) in the NgR1 KO-CCI group died after PTZ test (epileptic seizure).
(DOCX)

S2 Table Mouse Revised Neurobehavioral Severity Scale of WT and NgR1 KO mice at 1, 3, 7 and 14 days after CCI. No statistically significant differences were observed between the WT+CCI and NgR1 KO+CCI groups at any time point (p >  0.05, two-way ANOVA).
(DOCX)

## Abbreviations

TBI: Traumatic brain injury
CCI: Controlled cortical impact
WT: Wild-type
NgR1: Nogo-66 receptor1
KO: Knockout
CNS: Central nervous system
i.p: Intraperitoneally
PTZ: Pentylenetetrazol
LTP: Long-term potentiation
mNSS-R: Mouse Revised Neurobehavioral Severity Scale
TEM: Transmission electron microscopy
EEG: Electroencephalogram
IF: Immunofluorescence
RMS: Root mean square;
min: , minutes;
MIPs: Maximum image projections
ROI: Regions of interest
PSD: Postsynaptic density
