# Supplementary figures and images for: NgR1 knockout increased neuronal excitability and altered seizure pattern in traumatic brain injury mice brain after PTZ-induced seizure
Source: PLoS One. 2025 Apr 15;20(4):e0321447. doi: 10.1371/journal.pone.0321447 (PMC11999111; doi:10.1371/journal.pone.0321447)

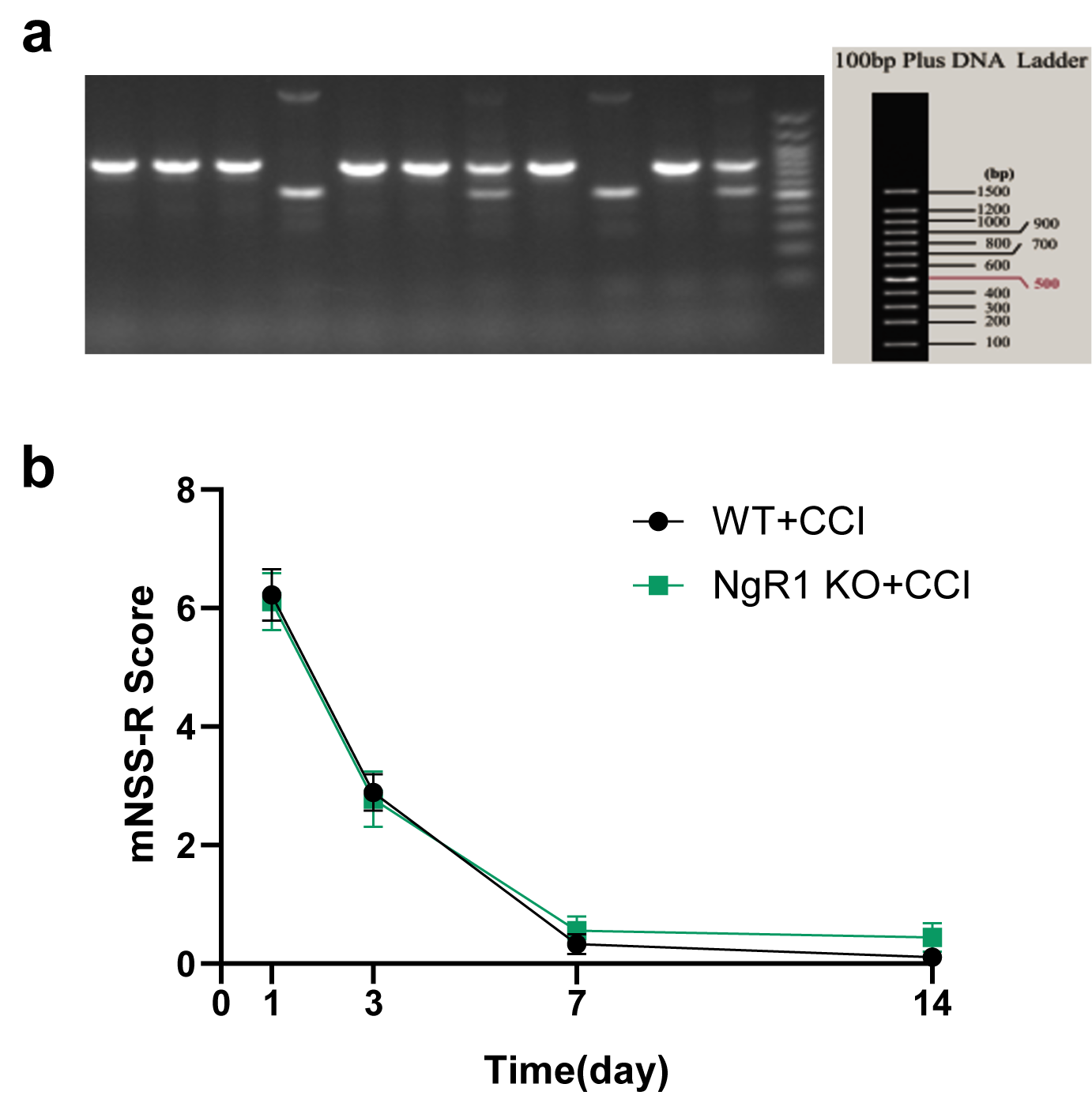

Supplement: S1 Fig — Among them, a single 512-bp band represents wild-type mice, the presence of both 512-bp and 735-bp bands indicates heterozygosity, and a single 735-bp band signifies homozygosity. (b)Mouse Revised Neurobehavioral Severity Scale of WT and NgR1 KO mice were performed at 1, 3, 7 and 14 days after CCI. (c) The results showed that the severity of neurological function loss was the highest at 1 day after traumatic brain injury. After that, the neurological function gradually improved. There was no significant difference in Neurobehavioral Severity Scale between the two groups (n = 9, two-way ANOVA). (TIF) [file pone.0321447.s001.tif]

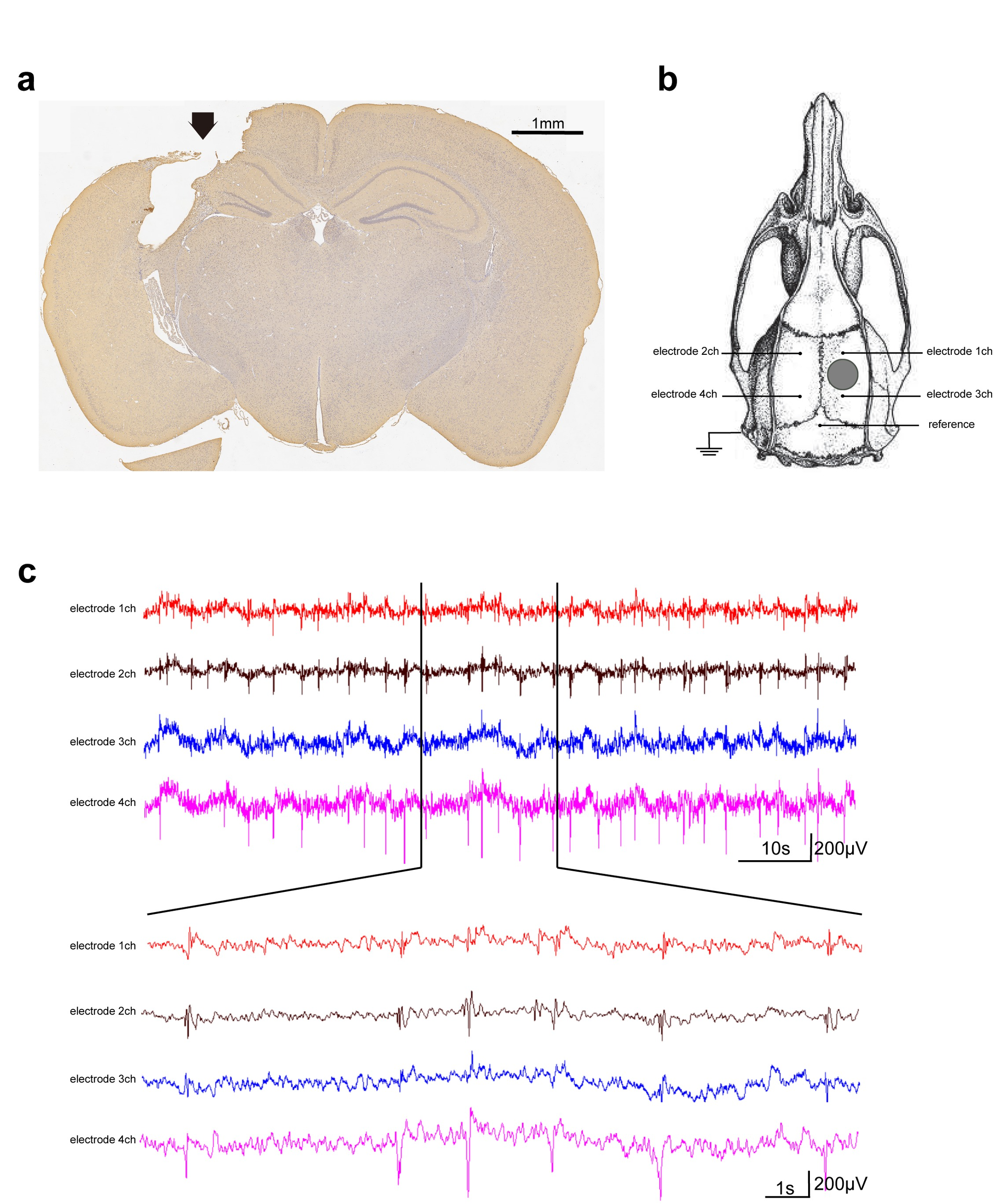

Supplement: S2 Fig — The black arrow indicates the injury site. (b) Diagram of cortical electrode implantation. (c) Primary EEG signal obtained from the 4 electrodes. Electrode 1ch and electrode 3ch represent ipsilateral electrodes (injured side of the brain). Electrode 2ch and electrode 3ch collected contralateral cortical EEG signals. Note that only the ipsilateral EEG data were used for the subsequent Morlet wavelet analysis and RMS power analysis. (TIF) [file pone.0321447.s002.tif]

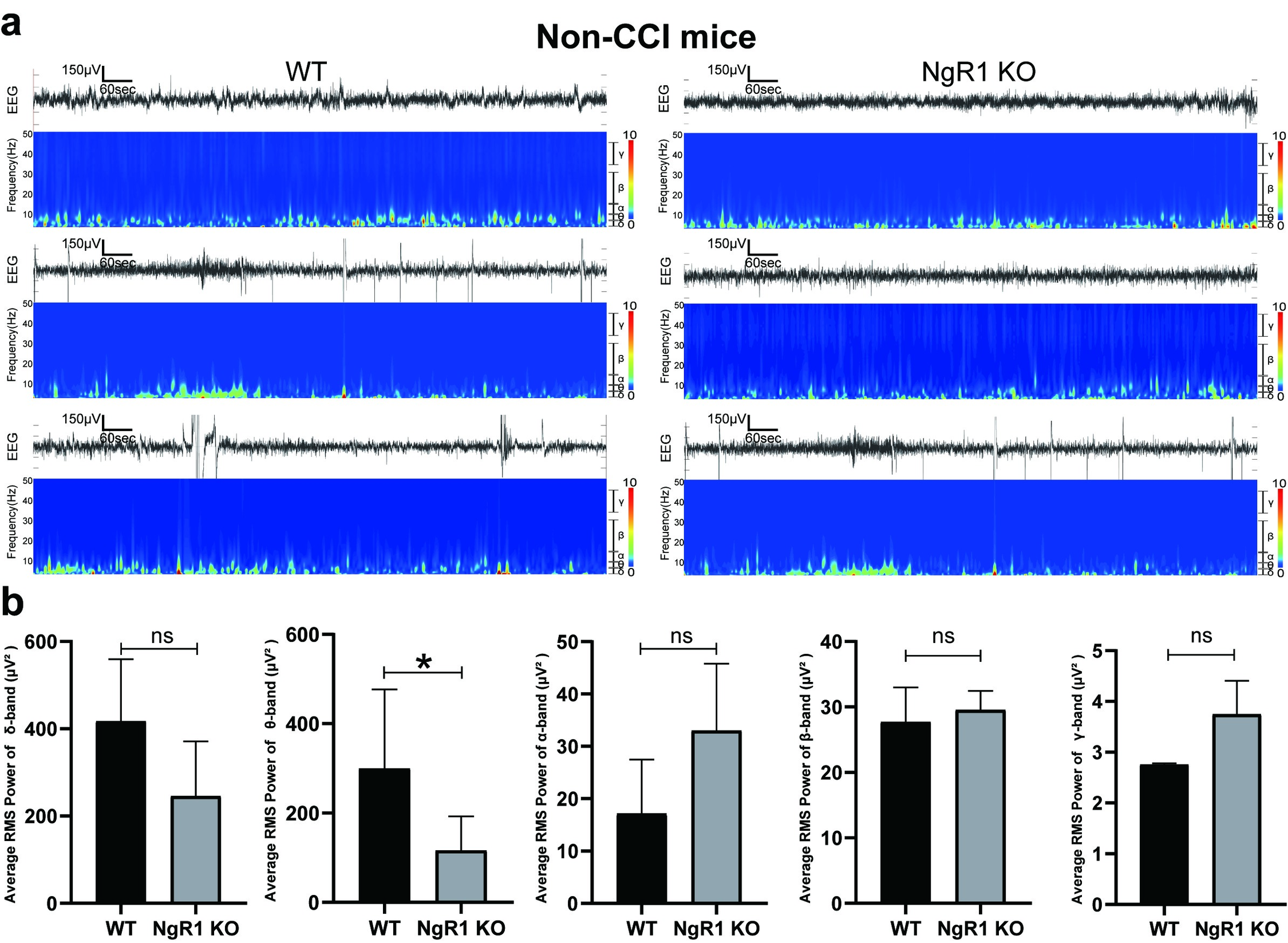

Supplement: S3 Fig — (a) The EEG signals and spectral heatmap of a representative WT mice and NgR1 KO mice are shown and the vertical axis of the spectral heatmap displays the frequencies, which are categorized into 5 bands (δ, θ, α, β, γ), with corresponding labels presented on the right vertical axis. In addition, on the right scale bar, warm colors represent higher power, and cool colors represent lower power. (a) From the range and concentration of warm colors in the background, it is evident that WT mice and NgR1 exhibit predominant brain electrical power at lower frequencies (δ, θ); (b) Power analysis including the δ, θ, α, β, γ bands in WT and NgR1 KO mice. The RMS analysis revealed that the baseline power in the δ, θ frequency bands of NgR1 KO mice was lower compared to WT mice. Conversely, the baseline power in the α, β and γ frequency bands was higher in NgR1 KO mice than in WT mice. However, there were no statistically significant differences observed in δ, α, β, and γ when considering the overall statistics. Only in the θ band did NgR1 KO mice exhibit lower baseline power compared to WT mice, with statistical significance. (ns, non-statistical significance; * P < 0.05). (TIF) [file pone.0321447.s003.tif]
