# Supplementary material for: NgR1 knockout increased neuronal excitability and altered seizure pattern in traumatic brain injury mice brain after PTZ-induced seizure
Source: PLoS One. 2025 Apr 15;20(4):e0321447. doi: 10.1371/journal.pone.0321447 (PMC11999111; doi:10.1371/journal.pone.0321447)
Supplement: S1 Table — The injury-related mortality rate within the 24 h post-CCI was 5.3% (1/19) in the WT-CCI group and 5.9% (1/17) in the NgR1 KO-CCI group, with both deaths attributed to subdural hematoma. Follow-up mNSS-R evaluations were performed on postoperative day 1、3、7、14 to assess neurobehavioral dysfunction. 1/16 mice (6.3%) in the NgR1 KO-CCI group died in 4–7days post-CCI (euthanized due to poor wound healing resulting in decapitation), and 1/18 mice (5.6%) in the WT-CCI group died in 8–14 days post-CCI (cause of death unknown). 1/15 mice (6.7%) in the NgR1 KO-CCI group died after PTZ test (epileptic seizure). (DOCX) [file pone.0321447.s004.docx]

# Supplementary Table 1 Mortality in WT and NgR1 KO mice after CCI and after PTZ test.

| Animal group | mortality of 24h post-TBI | mortality of 4-7days post-CCI | mortality of 8-14 days post-CCI | mortality after PTZ test (epileptic seizure) |
| --- | --- | --- | --- | --- |
| WT | 1/19(5.3%) | 0/18(0%) | 1/18 (5.6%) | 0/17(0%) |
| NgR1 KO | 1/17(5.9%) | 1/16(6.3%) | 0/15 (0%) | 1/15(6.7%) |
