# Supplementary material for: NgR1 knockout increased neuronal excitability and altered seizure pattern in traumatic brain injury mice brain after PTZ-induced seizure
Source: PLoS One. 2025 Apr 15;20(4):e0321447. doi: 10.1371/journal.pone.0321447 (PMC11999111; doi:10.1371/journal.pone.0321447)
Supplement: S2 Table — No statistically significant differences were observed between the WT+CCI and NgR1 KO+CCI groups at any time point (p > 0.05, two-way ANOVA). (DOCX) [file pone.0321447.s005.docx]

# Supplementary Table 2 Mouse Revised Neurobehavioral Severity Scale of WT and NgR1 KO mice at 1, 3, 7 and 14 days after CCI.

| Time (day) | WT+CCI (n=9)  Mean±SEM | NgR1 KO+CCI (n=9)  Mean±SEM | *P* Value |
| --- | --- | --- | --- |
| 1 | 6.22±0.43 | 6.11±0.48 | 0.97 |
| 3 | 2.89±0.31 | 2.78±0.46 | 0.97 |
| 7 | 0.33±0.17 | 0.56±0.24 | 0.95 |
| 14 | 0.11±0.11 | 0.44±0.24 | 0.93 |
